# Alumni of Southern Medical College

**Published:** 1892-02

**Authors:** 


*To the Alumni of the Southern Medical College:*

The undersigned, after due deliberation upon the matter, have decided to issue this call to all the graduates of the Southern Medical College to meet in Atlanta, Ga., March 3d, at 9 o'clock a. m., in the halls of the college building, to organize an alumni association, such association to meet each year, and during its session to be represented in the regular annual commencement exercises by an orator appointed by the association the previous year.

The association shall have for its object the social and professional advancement of its members.

In view of the fact that graduates of other institutions of note have formed similar organizations, we issue this formal call.

On the evening of the 3d, the first of a series of annual banquets will be held in conjunction with that of the Southern Medical Society. All wishing to secure plates for same will please send their names by February 20th to Dr. John W. Price, West End, Atlanta, Ga.

(Signed): THOS. CARR AVARY,
JNO. W. PRICE,
C. C. GREENE,
A. AVARY,
W. B. PARKS,
A. DAWSON,
N. O. HARRIS,
C. E. JOHNSON,
E. VAN GOIDTSNOVEN.

